# Women and the early *Journal of Physiology*

**DOI:** 10.1113/jphysiol.2014.288258

**Published:** 2015-01-15

**Authors:** Tilli Tansey

**Affiliations:** ^1^ School of HistoryQueen Mary University of LondonMile End RoadLondonE1 4NSUK

It was 1913, and one of those rare occasions when Ernest Starling disagreed with his fellow UCL Professor of Physiology and brother‐in‐law, William Bayliss, about a physiological issue. The question related to the admission of women to membership in The Physiological Society. Founded in 1876 as a dining club, the Society had never explicitly excluded women. But habit and practice had meant that women did not attend its meetings as members, although they had certainly contributed to some of its meetings, which from December 1880 included live scientific demonstrations and the reading of papers. The centrality of the dinner, which followed the Society's scientific proceedings and which routinely included live animal experiments at that time, is emphasized by Starling's view, when considering women as possible members, that ‘it would be improper to dine with ladies smelling of dog – the men smelling of dog that is’ (Evans, 1964). Despite the reservations of Starling and other colleagues, a ballot proposed by J. S. Haldane and J. N. Langley resulted, in mid‐1914, in an overwhelming vote to allow women members (although there was a substantial minority who wanted them barred from the dinner), and a formal resolution was adopted by the AGM in January 1915 to allow women to be eligible for membership, the first being elected later that year: Florence Buchanan, Winfred Cullis, Ruth Skelton, Sarah Sowton, Constance Leetham Terry and Enid Tribe.

These six women were all active physiologists, several having given communications to the Society or published in either *The Journal of Physiology* (*The Journal*) or the *Quarterly Journal of Experimental Physiology* (*QJEP*, now *Experimental Physiology*) or both, although little is yet known about their lives (see Tansey, 1993). It was probably the proposal of Florence Buchanan for membership of the Society by J. S. Haldane around 1912 that initially stimulated the formal debate outlined above. Working from the Physiological Laboratory, Oxford, Buchanan delivered at least 10 communications to the Society prior to her election, in addition to publishing full papers in both *The Journal* and *QJEP*. Indeed, she had two papers in the first volume of the latter, including the very first paper of all (Buchanan, 1908a,b). Her contributions to the Society and *The Journal* included electrophysiological work on skeletal muscle fibres and comparative studies of cardiac function, including cardiovascular assessments of the effects of hibernation (e.g. Buchanan, 1899, Buchanan, 1901; see also Mitchell, 2013).

Sarah Sowton had a rather diverse scientific career. Before her election, she had studied the effects of carbon dioxide on skeletal and cardiac muscle function at St Mary's Hospital Medical School in London with Augustus Waller, who was well known for his support of women, including his wife (see e.g. Waller, 1914). A little later, Sowton collaborated with Charles Sherrington, especially on the effect of chloroform on spinal reflexes (Sherrington & Sowton, 1911, 1915) Following her election, she was the co‐author of work on anaphylaxis with the husband and wife team of A. S. and H. G. Leyton (formerly Grunbaum), Helen Grunbaum also being a regular contributor to the Society (see Table 1). By 1928, Sowton was working for the Industrial Fatigue Research Board (Sowton & Myers, 1928). Joseph Barcroft from Cambridge proposed both Ruth Skelton, from University College London, and Constance Leetham Terry (later Oppenheimer), from the London School of Medicine for Women, both women then working in cardiovascular physiology. Leetham had published in *The Journal*, but Skelton appears not to have done so, her major paper in *The Journal* appearing some years after her election to the Society (Leetham, 1913; Skelton, 1921). Barcroft was also the proposer of Enid Tribe, a lecturer in Histology at the London School of Medicine for Women (see e.g. Tribe, 1914). One of Tribe's first papers was in collaboration with the sixth woman elected in 1915, Winifred Clara Cullis (Cullis & Tribe, 1913), who became the most distinguished member of this group. Cullis had been appointed a demonstrator in physiology at the London School of Medicine for Women in 1901, following training in Cambridge and research work in the Physiological Laboratory there under J. N. Langley. She was promoted to lecturer 2 years later, and in 1908 was awarded a University of London DSc (Cullis, 1908). In 1912, she became Reader and Head of Department, before achieving the title and status of Professor of Physiology in 1919. Her research work ranged widely; before 1915, full papers appeared in *The Journal* on urine secretion, gut gas metabolism, coronary vessel innervation, cardiac innervation and atrioventricular node function (Brodie & Cullis 1906, 1908a,b, 1911; Brodie, Cullis & Halliburton 1910; Cullis 1906; Cullis & Dixon 1911). Cullis was a physiological pioneer in other ways; she was the first woman to serve on The Physiological Society's Committee, from 1918 to 1925, and also the first woman to preside at a Meeting of the Society in 1920.

**Table 1:** **Women authors (of communications, demonstrations and full papers) in *The Journal of Physiology* 1878–1915**

Harriet Bills	Marie Krogh
Julia Brinck	Janet E. Lane‐Claypon
A. Miriam Bruce	Constance Leetham
Florence Buchanan	Doris L. Mackinnon
Elizabeth Cooke	Marion I. Newbiggin
Harriette Chick	Dorothy Norris
Winifred Cullis	Emily Nunn
Dorothy Dale	Helen Perkins
Florence Durham	Myra E. Pollard
Elizabeth E. Eaves	Agnes Ellen Porter
Beatrice Edgell	C. B. Sanders
Florence Eves	Edith R. Saunders
Mabel P. Fitzgerald	Ida Smedley
Laura Elizabeth Forster	Sarah C. M. Sowton
Marion Greenwood	Mary Christine Tebb
Helen G. Grunbaum	Florence D. Thompson
Gladys Hartwell	Enid M. Tribe
Evelyn E. Hewer	May Tweedy
A. Muriel Hill	Nora Tweedy
Annie Homer	Mary D. Waller
Lily H. Huie	Edith G. Willcock
Helen P. Kemp	

But there were far more than these six women contributing to British physiology prior to 1915. In addition to full papers, several contributed communications and demonstrations to the Society and some had even attended a hallowed dinner (Tansey, 1993). By 1915, more than 50 individual women can be identified as having been contributors to *The Journal* since its first volume in 1878 and to the *QJEP* since its inauguration in 1908 (see Tables 1 and 2).

As noted above, Florence Buchanan was the author of the very first paper in the *QJEP*. The first volume of the privately owned *Journal* (1878/9) also included two additional women authors, both American. One, Harriet Bills, was listed only as ‘assisting’ V. C. Vaughan (Fig. 1), although the second, Emily Nunn, was clearly identified as the sole author of her work (Nunn, 1878; Vaughan & Bills, 1879). Then a Lecturer in Biology at Wellesley College, Boston, Nunn's introductory sentence makes clear that she was already well connected with British physiologists: ‘[A]t Dr Ringer's desire, I undertook, under Dr Foster's guidance, an examination of the condition of the epidermis of frogs after poisoning by arsenic and by antimony…’ (Nunn, 1878). Nunn was later recommended to the prestigious Cambridge Table at the Naples Zoological Station by Thomas Henry Huxley and Michael Foster (Creese, 1998).

**Figure 1:**
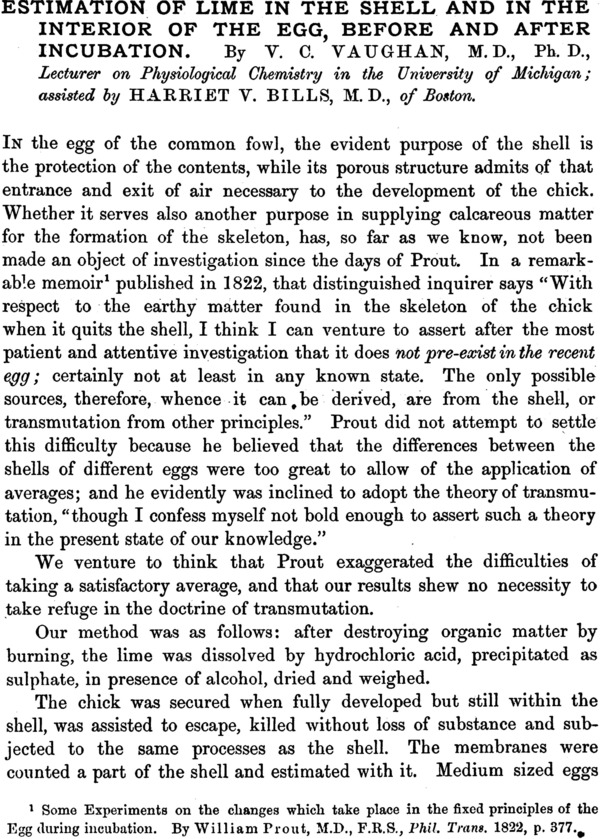
Title of paper by V C Vaughan & Harriet Bills (1879)

The lists in Tables 1 and 2 contain many names little known, if at all, to modern physiologists. Yet many were very well known in their lifetimes, and they deserve to be remembered for their work and contributions. Here, I shall mention only a few. Janet Lane‐Claypon's early biochemical work in Ernest Starling's laboratory at UCL led to a lifetimes’ interest in nutrition, public health and the development of epidemiological studies of maternity and child welfare (Lane‐Claypon, 1905; Lane‐Claypon & Schryver, 1904). Likewise, Harriette Chick (later Dame Harriette) started her career studying basic biochemical processes, which were published in *The Journal* (Chick & Martin, 1910, 1911, 1912a,b). She became a notable nutritionist, especially renowned for her work on rickets and malnutrition. Elected to The Physiological Society in 1918, she was, almost 50 years later, elected the first woman Honorary member of the Society in 1967. May Tweedy also became a renowned nutritionist, better known under her married name of May Mellanby (Fig. 2), often working with her husband Edward Mellanby on the role of vitamin D in the prevention of rickets. Working in Starling's UCL laboratory, she first published on gastric secretion in *The Journal*, working with J. S. Edkins, who later became her brother‐in‐law (Edkins & Tweedy, 1909). Her sister Nora started her physiological career at Bedford College (then a women's college of the University of London), publishing a study of the effects of exercise on fellow students whilst still an undergraduate (Hartwell & Tweedy, 1913). As Nora Edkins, much of her experimental work was in gastric and intestinal physiology (e.g. Edkins & Murray, 1924, 1926). Her younger colleague, Margaret Murray, becoming the first woman member of the Editorial Board of *The Journal* in 1949. Like Winifred Cullis, she influenced generations of women students, being promoted to the headship of the Department of Physiology at Bedford College in 1929, although unlike Cullis she was denied the title of Professor.

**Table 2:** **Women authors (of full papers) in *Quarterly Journal of Experimental Physiology* (1908–1915)**

Williamina Abel	Caroline M'Gill
Barbara Ayrton	Marjory MacNaughton
Florence Buchanan	A. E. Porter
Lyda May Degener	M. Christine Tebb
Alice L. Embleton	Frances M. Tozer
Frances M. Huxley	Rosalind Wulzen
Jessie Luella King	

**Figure 2:**
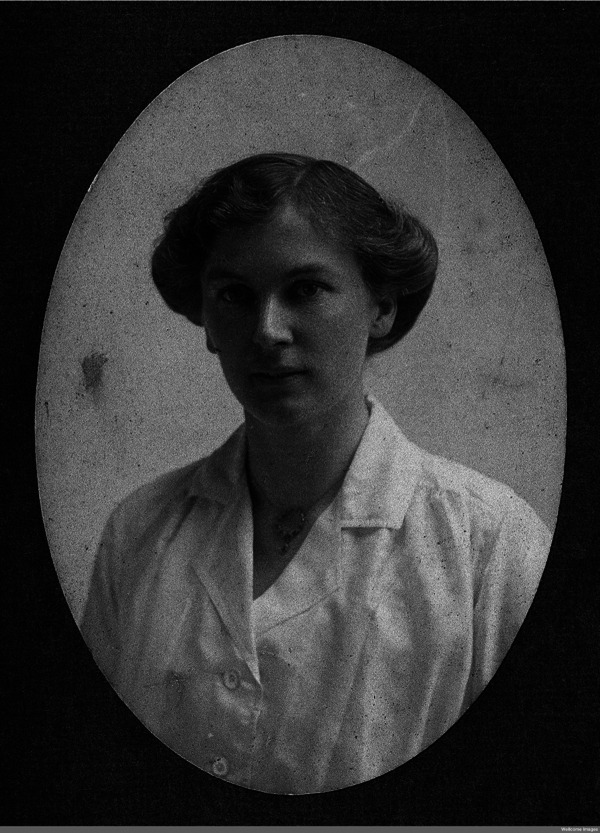
**May Mellanby** Photograph by Langfiér Ltd. From the Wellcome Library, London, reproduced with permission.

Now, a century after the election of the first women to The Physiological Society, it seems strange to read of a time when it was difficult for them to be full members of a scientific society. It does seem clear, however, from an examination of the early volumes of *The Journal*, that there was never any discrimination against women authors. Indeed a substantial cohort of women authors has been revealed, and their publications in *The Journal*, and later the *QJEP*, contributed to several distinguished careers, many sadly now forgotten.

## Additional information

### Competing interests

None declared.
